# Aortic knuckle calcification

**DOI:** 10.11604/pamj.2023.44.168.31991

**Published:** 2023-04-12

**Authors:** Surya Besant Natarajan, Krishna Prasanth Baalann

**Affiliations:** 1 Department of Community Medicine, Sree Balaji Medical College and Hospital, Bharath Institute of Higher Education and Research, Shankar Nagar, Chromepet, Chennai, Tamil Nadu 600044, India

**Keywords:** Hypertension, rheumatic fever, dyspnea

## Image in medicine

Aortic knuckle calcification is a condition where there is deposit of calcium in aortic valve causing reduced blood flow through the same. The condition usually presents in old age as a complication of rheumatic heart disease or Infective Endocarditis. Risk factors include old age, raised blood pressure and coronary heart disease. A 60-year-old female came with chief complaints of breathing difficulty for one month (grade 2 dyspnea) along with chest pain for two days. There was a history of rheumatic fever five years ago and was treated with a ten days course of penicillin. On examination, her blood pressure was found to be 150/100mmHg. Chest X-ray was taken and it showed aortic knuckle calcification. The patient was started on anti-hypertensive drugs and rheumatic fever prophylaxis and was planned for surgical valve replacement after 3 months.

**Figure 1:**
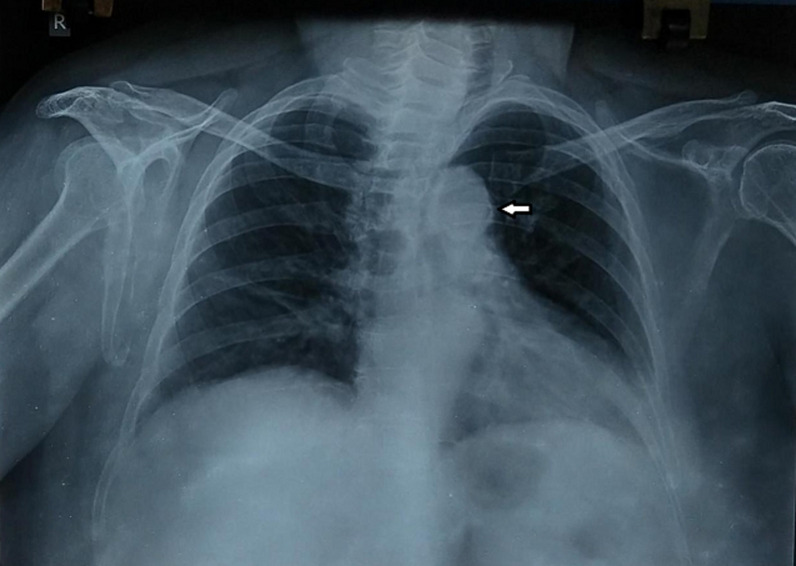
radio-opaque mass over the hilar region: aortic knuckle calcification

